# Tubular retractor-assisted minimally invasive parafascicular approach for dermoid cyst

**DOI:** 10.1093/jscr/rjaf066

**Published:** 2025-02-19

**Authors:** Keng Siang Lee, Nida Kalyal, Engelbert Mthunzi, Francesco Marchi, Ali Elhag, Istvan Bodi, Ranjeev Bhangoo, Francesco Vergani, Keyoumars Ashkan, Richard Gullan, Jose Pedro Lavrador

**Affiliations:** Department of Neurosurgery, King’s College Hospital Foundation Trust, London SE5 9RS, United Kingdom; Department of Basic and Clinical Neurosciences, Maurice Wohl Clinical Neuroscience Institute, Institute of Psychiatry, Psychology and Neuroscience (IoPPN), King's College London, London SE5 9RX, United Kingdom; Department of Neurosurgery, King’s College Hospital Foundation Trust, London SE5 9RS, United Kingdom; Department of Neurosurgery, King’s College Hospital Foundation Trust, London SE5 9RS, United Kingdom; Department of Neurosurgery, King’s College Hospital Foundation Trust, London SE5 9RS, United Kingdom; Department of Neurosurgery, Neurocenter of Southern Switzerland, Ente Ospedaliero Cantonale, Lugano 6900, Switzerland; Department of Neurosurgery, King’s College Hospital Foundation Trust, London SE5 9RS, United Kingdom; Department of Clinical Neuropathology, Academic Neuroscience Centre, King’s College Hospital Foundation Trust, London SE5 9RS, United Kingdom; Department of Neurosurgery, King’s College Hospital Foundation Trust, London SE5 9RS, United Kingdom; Department of Neurosurgery, King’s College Hospital Foundation Trust, London SE5 9RS, United Kingdom; Department of Neurosurgery, King’s College Hospital Foundation Trust, London SE5 9RS, United Kingdom; Department of Neurosurgery, King’s College Hospital Foundation Trust, London SE5 9RS, United Kingdom; Department of Neurosurgery, King’s College Hospital Foundation Trust, London SE5 9RS, United Kingdom

**Keywords:** dermoid cyst, minimally invasive, neurosurgery, parafascicular surgery, tubular retractor, tractography

## Abstract

Intracranial dermoid cysts are benign lesions that may be diagnosed incidentally or present symptomatically due to mass effect—focal neurological deficits, seizures and/or hydrocephalus—or chemical meningitis secondary to spontaneous rupture. The use of tubular retractors in minimally invasive parafascicular surgery (tsMIPS) has been described extensively as a technique to preserve neurological function whilst safely maximizing the extent of resection. The authors present the first use of the tsMIPS approach for removal of a dermoid cyst in a 68-year-old female who presented with abulia and seizures due to a large Sylvian fissure dermoid cyst. This approach minimized trauma to surrounding cortical–subcortical structures, as supported by connectome analyses, without sacrificing visualization of the operative field. Additionally, itavoided manipulation of the lenticulostriate arteries attached to the walls of the dermoid cyst. The use of an endoscope ensured complete drainage of the cyst components and therefore the effectiveness of the procedure.

## Introduction

Intracranial dermoid cysts are rare, benign lesions of embryological origin, accounting for <0.5% of all intracranial tumors [1–3]. They are typically located in the midline [3], and as part of their natural history, enlarge slowly and accumulate thick, yellowish material composed of desquamated epithelium, sebaceous gland secretions, fat, and hair [2]. These cysts may be diagnosed as incidental findings or as symptomatic lesions related to mass effect over surrounding brain or after their rupture. In cases of rupture, dissemination of intracystic contents into the subarachnoid space [2], can lead to chemical aseptic meningitis or new-onset seizures.

The application of tubular retractor-assisted minimally invasive surgery (tsMIPS) has been well documented in recent decades, especially for deep seated brain lesions [4, 5]. To our knowledge, this is the first reported case of an intracranial dermoid cyst removal employing the tsMIPS approach [6].

## Case report

### History and examination

**Figure 1:**
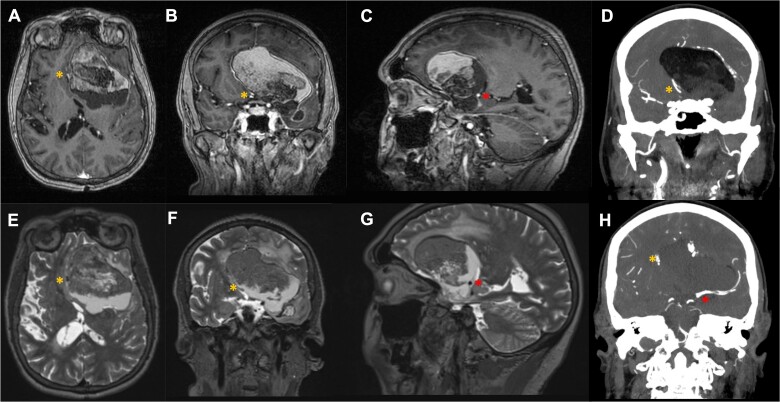
Pre-operative MR T1 post gadolinium [axial (A), coronal (B), sagittal (C)], coronal CT angiogram (D, H) and T2 [axial (E), coronal (F), sagittal (G)] demonstrating the vascular relationship of the left Sylvian fissure-centered dermoid with the anterior cerebral artery (orange *) and middle cerebral artery (red *) seen displaced by the lesion. There is surrounding edema (E,F,G) and heterogeneity in contrast enhancement (A,B,C), as well as a fluid level (A,B,C,E,F,G).

A 68 year-old female was diagnosed with a large heterogenous dermoid cyst centered in the left Sylvian fissure, extending toward the subfrontal space, following theonset of generalized tonic–clonic seizures (Fig. 1). Her seizures were controlled with antiepileptic drugs and she remained stable for 10 years. In the months preceding her referral to our neurosurgical unit, she developed focal neurological symptoms related to the mass effect caused by the lesion, including speech disturbances, global frontal lobe dysfunction with abulia and loss of urinary sphincter control.

### Operative workflow and intraoperative findings

**Figure 2:**
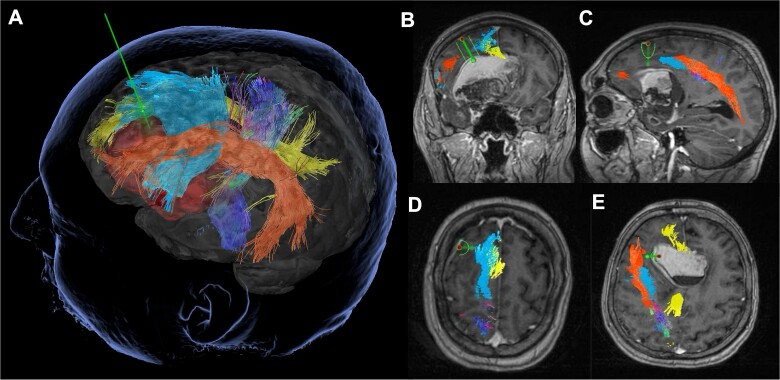
Tubular retractor-assisted minimally invasive parafascicular surgery approach: Pre-operative tractography demonstrating the relationship of the left Sylvian fissure-centered dermoid to the corticospinal tract (dark blue), frontal-aslant tract (light blue), cingulate (yellow) and arcuate fasciculus (orange).

Preoperatively, a contrast-enhanced MRI with diffusion sequences for tractography dissection was performed. StealthStationS8© (Medtronic Inc., Louisville, CO, USA) was used for pre-surgical planning—tractography dissection, tumor modeling, and trajectory planning. The trajectory planning was utilized to maximize subcortical tract preservation, avoiding the bulk of the fronto-aslant tract (FAT), cingulate, uncinate fasciculus, and arcuate fasciculus. The inferior fronto-occipital fasciculus could not be dissected (Fig. 2).

**Figure 3:**
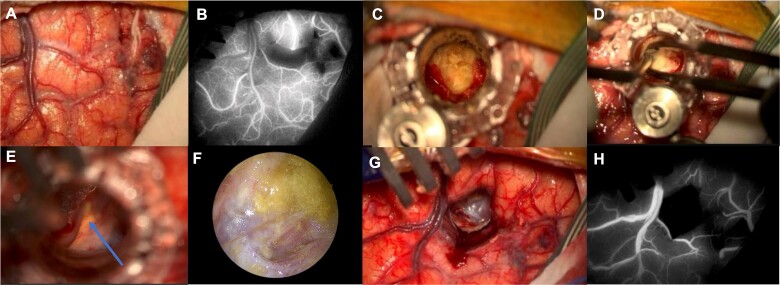
Surgical workflow: Left superior frontal sulcus dissection (A) with preservation of the cortical vasculature of the adjacent gyri shown by the ICG with the infrared image on the Zeiss KINEVO 900© microscope (B); introduction of the NICO BrainPath©, dissection of the dermoid capsule and visualization of the dermoid contents (C); the contents of the cyst were removed (D) and anatomical structures such as the optic nerve (blue arrow) were visualized through the dermoid cyst capsule (E); the endoscope was used at the end of the drainage to visualize remnants of the cyst and they were further washed and removed (F); the tubular retractor was removed (G) and the preservation of the cortical vasculature after the procedure was verified with the use of ICG (H).

After skin incision, craniotomy and dural opening, the arachnoid overlying the entry point on the superior frontal sulcus was divided. Indocyanine green (ICG) angiography confirmed the integrity of the vasculature in the adjacent gyri after arachnoid dissection. After sulcal split, a 13.5 × 60 mm tubular retractor (Nico©BrainPath) was gently inserted along the previously planned surgical trajectory, into the tissue overlying capsule. This capsule was opened, with its contents removed. An endoscope (ZeissQevo©) was inserted for intra-capsular inspection which confirmed no remnant (Fig. 3 and Video 1).

### Histopathological findings

**Figure 4:**
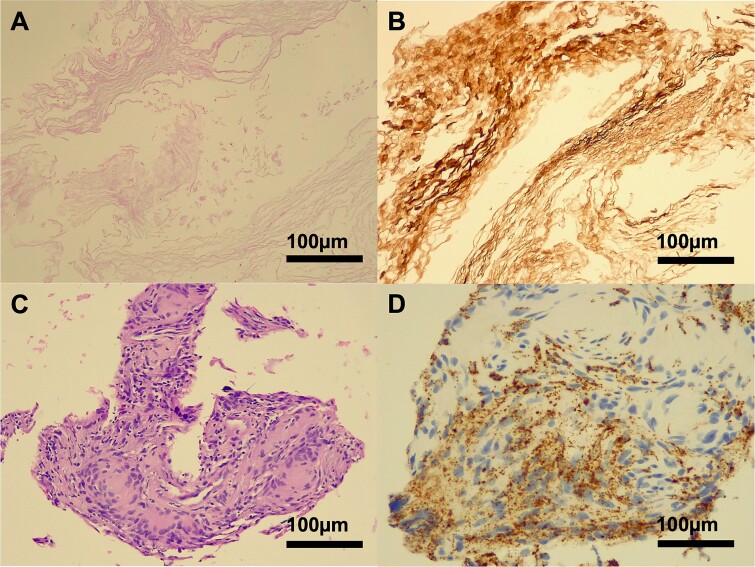
Histopathology of a ruptured keratinous cyst. Hematoxylin-eosin (H&E) staining demonstrated a lamellar arrangement of necrotic keratin (A), confirmed by immunohistochemistry (B) for pan-cytokeratin (MNF116). A cluster of foreign body type macrophages was identified on H&E staining (C), with CD68 positivity (D). No viable epithelial lining was present in the biopsy specimen.

Microscopic examination revealed a lesion composed predominantly of cells with necrotic keratin debris positive for cytokeratin. The cellular component included giant cells and macrophages positive for CD68, consistent with a previous rupture of an epidermoid/dermoid cyst (Fig. 4).

### Outcome and follow-up

**Figure 5:**
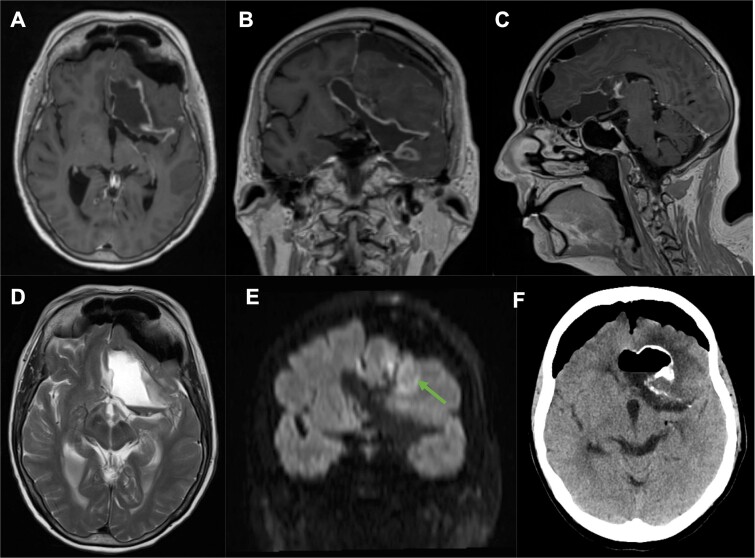
Post-operative MR T1 post gadolinium [axial (A), coronal (B), sagittal (C)], axial T2 (D), coronal DWI B1000 (E) and axial CT (F) confirmed complete evacuation of the dermoid cyst contents with the capsule preserved *in situ*.

Postoperative imaging showed complete drainage of the dermoid cyst with the capsule left *in-situ*. There were no surgical complications (Fig. 5).

**Figure 6:**
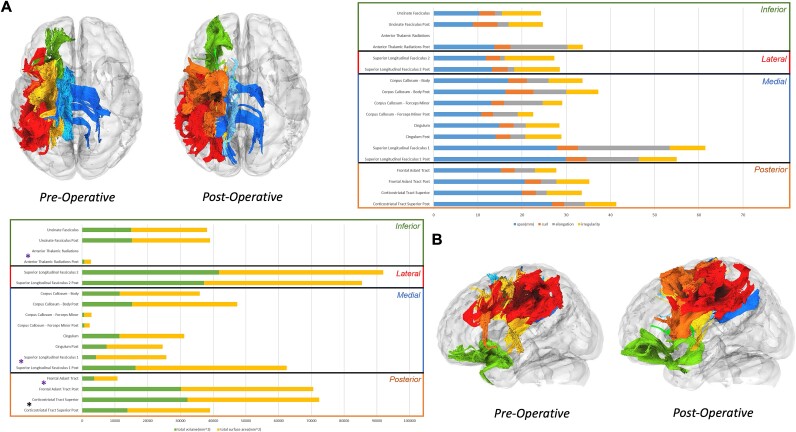
Tractography analysis: a tractography analysis of the peritumoral tracts and those located around the surgical corridor was performed before and after tumor resection. An improvement in the morphology of the frontal aslant tract and SLF I (both volume and area) and a successful dissection of the anterior thalamic radiations was verified after surgery alongside with a deterioration of the superior corticalstriatal tract (A). With regards to the microstructural metrics, we observed no significant differences between the preoperative and the postoperative tractography of the analyzed tracts.

**Figure 7:**
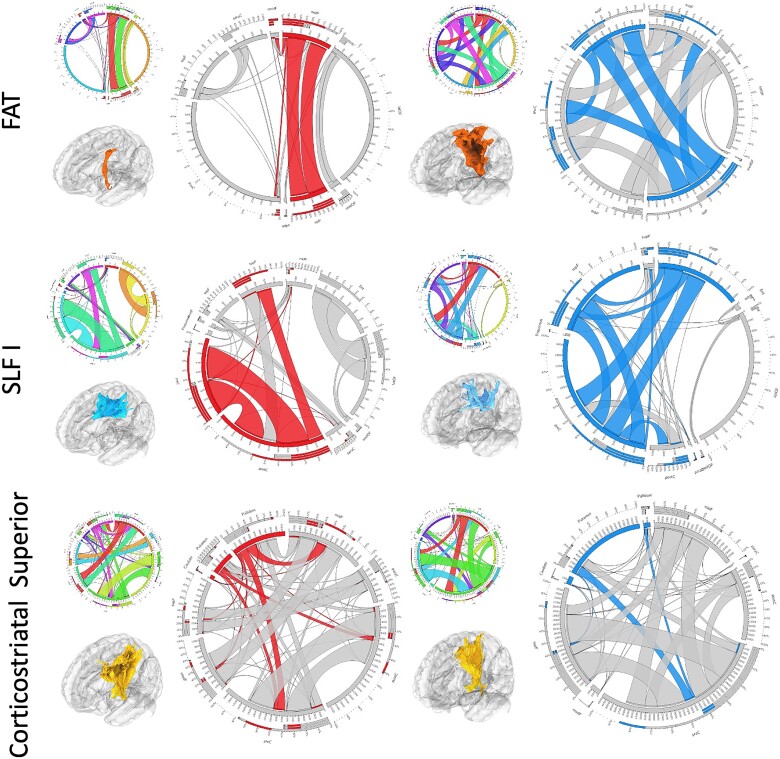
Connectivity analysis: a connectivity analysis of the tracts with changes in the morphometry was performed. We observed an improvement of the connectivity of both fronto-aslant tract (FAT) and SLF I around the surgical tract—FAT showed increased connectivity in the superior frontal gyrus and the SLF I in the superior and middle frontal gyri. For the corticostriatal gyrus, we observed decrease connectivity to the basal ganglia. Red—predominant preoperative connectivity; blue—predominant postoperative connectivity

Post-operative diffusion imaging allowed for tractography and connectivity analysis of the peritumoral and peri-surgical corridor areas. An improvement in the morphometry (particularly area and volume of the tract) of the FAT, superior longitudinal fasciculus I (SLF I) and successful dissection of the anterior thalamic radiations was observed after surgery. There was a deterioration of these same metrics for the superior corticostriatal tract. There were no significant changes in the fractional anisotropy and diffusivity metrics across all analyzed tracts (Fig. 6). Connectivity analysis of the tracts that showed changes in the morphometry suggested a normalization of the connectivity patterns of the tracts, particularly in regions around the tumor and surgical corridor (Fig. 7).

The patient was discharged three days after surgery. At one-month follow-up, she was able to perform daily activities with mild support. Her speech function had normalized although she exhibited mild dysfunction in speech fluency.

## Discussion

As supported by our systematic review (registration number: CRD42024589522) (Supplementary Tables 1 and 2), this is the first study to assess the use of the tubular retractor system for the removal of an intracranial dermoid cyst [7].

Surgeries for deep-seated lesions are challenging because creating a corridor and observing the interface between lesions and normal brain tissue are difficult [8]. To circumvent these problems, many brain retraction systems combined with a microscope or endoscope have been developed [4, 5]. However, concerns surrounding excessive brain retraction pressures have been raised—the threshold for ischemia and contusion are estimated to be <30 mmHg [9]. The consequent danger of edema and brain infarction has been argued [9–11]. Additionally, systemic intraoperative factors, such as blood loss, acidosis, hypotension, and metabolic abnormalities, may further elevate the risk of cerebral ischemia induced by a brain retractor [4, 5, 12]. Subsequently, tubular retractor systems have been suggested as an alternative. This system can distribute the forces of retraction evenly and reduce brain retraction pressure to normal surrounding tissue—<10 mmHg [9–11]—compared with traditional retractors [4, 5, 12, 13], a level lower than the critical threshold for cerebral ischemia suggested by Rosenorn [9–11].

The concept of tubular retractor was proposed more than three decades ago, and their efficacy and safety have been reported numerously compared with traditional blade retractors that may cause asymmetric tension on brain tissue. The tubular retractor, specifically designed for neurosurgical applications, has been widely used for the removal of intraparenchymal or intraventricular tumors, hemorrhage, and foreign bodies [4, 5, 12, 13]. Its use for access to deep extra-axial lesion is controversial as it would imply a transparenchymal approach instead of arachnoid dissection and parenchymal retraction. However, tractography and connectivity analysis suggest a minimal footprint on cortical–subcortical tissue. Findings include a decrease in volume and total area of the superior corticostriatal tract while preserving the microstructural metrics (fractional anisotropy and diffusivity metrics) in the analyzed tracts. Additionally, an improvement in both FAT and SLF I volume and area was observed along with a normalization of the connectivity profile of these tracts toward.

Using TsMIPS with neuronavigation can improve precision and safety in surgery by creating a corridor that spares eloquent cortical–subcortical structures and mitigates against the risks associated with a conventional trans-Sylvian approach on the dominant hemisphere. This approach avoids brain retraction over eloquent language structures, reduces the risk of vasospasm, and vascular injury related with the arachnoid and vascular dissection. This is particularly the case in lesions where the vessels are adherent to the capsule as they were in this case [4, 14]. Complete resection of dermoid cysts is significantly associated with the lower risk of recurrence (recurrence rates between 0% and 26% reported) [15]. However, in elderly patients, such as in this 68 year old, the balance between a radical resection to prevent recurrence versus the risk of vascular injury and significant ischemia in the dominant hemisphere becomes a crucial consideration. 

Our study provides preliminary insights into the safety and effectiveness of tsMIPS for intracranial dermoid cysts [7]. However, we do not advocate for its indiscriminate use. As mentioned before, a careful risk—benefit assessment when comparing with conventional approaches—adherence to critical neurovascular structures and the risk of recurrence during expected lifetime—should be conducted by the surgical team and fully discussed with the patient.

## Conclusion

tsMIPS is a potential alternative when approaching large Sylvian dermoid cysts in elderly patients. This technique allows for functional tissue preservation, minimizes brain retraction and vascular dissection and enhances cyst content removal when assisted by an endoscope.

## Supplementary Material

Supplementary_dermoid_cyst_tubular_retractor_rjaf066

Final_video_with_endoscope_JPL_compressed_rjaf066
