# Supplementary material for: Tubular retractor-assisted minimally invasive parafascicular approach for dermoid cyst
Source: J Surg Case Rep. 2025 Feb 19;2025(2):rjaf066. doi: 10.1093/jscr/rjaf066 (PMC11837332; doi:10.1093/jscr/rjaf066)
Supplement: Supplementary_dermoid_cyst_tubular_retractor_rjaf066 [file supplementary_dermoid_cyst_tubular_retractor_rjaf066.docx]

Supplementary Table 1. Search strategy used for the three electronic databases 10 Sept 2024

| **EMBASE search** | | **0 articles** |
| --- | --- | --- |
| No. | Search term | |
| **Tubular retractor concept** | | |
| 1 | exp tubular retractor/ | |
| 2 | Tubular retractor.tw. | |
| 3 | 1 or 2 | |
| **Intracranial dermoid cyst concept** | | |
| 4 | exp dermoid cyst/ | |
| 5 | Dermoid cyst.tw | |
| 6 | 4 or 5 | |
| 7 | exp brain/ | |
| 8 | exp intracranial tumor/ | |
| 9 | 7 or 8 | |
| 10 | 6 and 9 | |
| **Combined concepts** | | |
| 11 | 3 and 10 | |

| **Medline search** | | **0 articles** |
| --- | --- | --- |
| No. | Search term | |
| **Tubular retractor concept** | | |
| 1 | Tubular retractor.tw. | |
| **Intracranial dermoid cyst concept** | | |
| 2 | exp dermoid cyst/ | |
| 3 | Dermoid cyst.tw | |
| 4 | 2 or 3 | |
| 5 | exp brain/ | |
| 6 | 4 and 5 | |
| **Combined concepts** | | |
| 7 | 1 and 6 | |

| **Cochrane Central Register of Controlled Trials (CENTRAL**) | | **0 articles** |
| --- | --- | --- |
| No. | Search term | |
| **Tubular retractor concept** | | |
| 1 | (tubular retractor):ti,ab,kw | |
| **Intracranial dermoid cyst concept** | | |
| 2 | MeSH descriptor: [dermoid cyst] explode all trees | |
| 3 | (dermoid cyst):ti,ab,kw | |
| 4 | #2 or #3 | |
| 5 | MeSH descriptor: [brain] explode all trees | |
| 6 | #4 and #5 | |
| **Combined concepts** | | |
| 7 | #1 and #6 | |

Supplementary Table 2. Inclusion and exclusion criteria used to select studies for the review

| Inclusion criteria | Exclusion criteria |
| --- | --- |
| Primary interventional or observational studies assessing management of dermoid cyst using the tubular retractor system | - Not written in English - Systematic reviews and meta-analysis, editorials, commentaries, opinion papers, letters, education papers, conference abstracts, protocols, reports, theses or book chapters - Treatment not tested in the clinical setting (e.g. lab based rather than clinical practice) - Non-human subjects (e.g. murine, porcine studies) - Outcomes not specific to dermoid cysts - Overlapping populations - Arm <3 patients |
